# Accurate and fast methods to estimate the population mutation rate from error prone sequences

**DOI:** 10.1186/1471-2105-10-247

**Published:** 2009-08-11

**Authors:** Bjarne Knudsen, Michael M Miyamoto

**Affiliations:** 1 CLC bio, 8200 Århus N, Denmark; 2 Department of Biology, Box 118525, University of Florida, Gainesville, Florida 32611-8525, USA

## Abstract

**Background:**

The population mutation rate (θ) remains one of the most fundamental parameters in genetics, ecology, and evolutionary biology. However, its accurate estimation can be seriously compromised when working with error prone data such as expressed sequence tags, low coverage draft sequences, and other such unfinished products. This study is premised on the simple idea that a random sequence error due to a chance accident during data collection or recording will be distributed within a population dataset as a singleton (i.e., as a polymorphic site where one sampled sequence exhibits a unique base relative to the common nucleotide of the others). Thus, one can avoid these random errors by ignoring the singletons within a dataset.

**Results:**

This strategy is implemented under an infinite sites model that focuses on only the internal branches of the sample genealogy where a shared polymorphism can arise (i.e., a variable site where each alternative base is represented by at least two sequences). This approach is first used to derive independently the same new Watterson and Tajima estimators of θ, as recently reported by Achaz [1] for error prone sequences. It is then used to modify the recent, full, maximum-likelihood model of Knudsen and Miyamoto [2], which incorporates various factors for experimental error and design with those for coalescence and mutation. These new methods are all accurate and fast according to evolutionary simulations and analyses of a real complex population dataset for the California seahare.

**Conclusion:**

In light of these results, we recommend the use of these three new methods for the determination of θ from error prone sequences. In particular, we advocate the new maximum likelihood model as a starting point for the further development of more complex coalescent/mutation models that also account for experimental error and design.

## Background

The population mutation rate (θ) remains one of the most fundamental parameters in genetics, ecology, and evolutionary biology [3-5]. This interest in θ derives from the fact that this parameter measures the effective size (*N*_*e*_) and whole-locus mutation rate (μ) of a population, which are of great importance in understanding its demography and history. Specifically, θ is a compound parameter that is calculated as the product of 2*pN*_*e*_μ (with *p *= 1 or 2 for haploids and diploids, respectively). Correspondingly, a number of alternative methods are available to estimate θ from a population sample of allelic sequences [6-8]. These alternative methods range from relatively simple summary statistics (moment methods) to full coalescent/mutation models. Indeed, the estimation of θ remains central to even the most complex coalescent/mutation models that are otherwise concerned with the determination of other population genetic parameters (e.g., for growth, migration, and recombination).

A population sample of sequences is obtained from interbreeding or potentially interbreeding individuals and is therefore usually associated with a small number of mutations [9-12]. Thus, when estimating θ from a population sample, sequence errors can pose a real problem, since their numbers can begin to approach or even surpass those for the mutations [13-19]. This problem becomes particularly acute when working with error prone data such as expressed sequence tags (EST), low coverage draft sequences, and other such unfinished products [20,21]. For example, an error rate of one mistake per every 500 nucleotides (e.g., as for an EST dataset obtained from single sequencing passes) will make a significant contribution to the observed variation among sequences that differ because of mutations by <1 to 2%. If uncorrected, such errors can lead to an inflated estimate of θ and even erroneous conclusions about the biology of their population [1,2,18,22].

**Figure 1:**
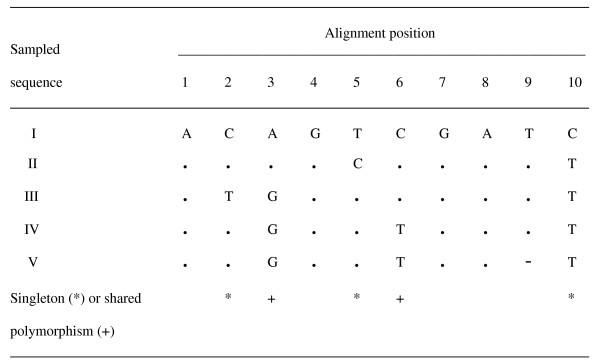
**Multiple sequence alignment for five hypothetical sequences as sampled from a single population**. Periods in sequences II-V refer to the same base as in I. The dash refers to a gap. Variable sites corresponding to singletons are marked with asterisks, whereas those representing shared polymorphisms are denoted with pluses.

Many sequence errors arise as random accidents that occur during the nucleic acid isolation, cloning/amplification, sequencing, and recording phases of a DNA sequencing study [11,23,24]. As chance events that are rare (even for error prone data), each of these random mistakes will most likely be limited to a single sequence, rather than repeated among two or more different ones within the population sample [1,15]. Thus, these random errors will most likely inflate the number of singletons within the dataset (i.e., polymorphic positions where one sampled sequence exhibits a unique base relative to the shared nucleotide of the others) (Figure 1). In contrast, these rare chance mistakes will make a much smaller contribution to the shared polymorphisms (i.e., variable sites where each alternative base is common to at least two different sampled sequences).

This study relies on the simple premise that the random mistakes of error prone sequences can be avoided by ignoring the singletons within their population sample. This strategy is first used to obtain independently the same new Watterson [25] and Tajima [26] estimators of θ, which were recently reported by Achaz [1] for error prone sequences. This approach is then implemented in the recent maximum likelihood (ML) model of Knudsen and Miyamoto [2], which incorporates various factors of experimental error and design with those for coalescence and mutation. By relying on only shared polymorphisms, these three new approaches allow for more accurate estimates of θ. However, this greater accuracy comes with a cost as singletons due to actual mutations are ignored along with those due to random errors. To assess this tradeoff, the three new methods are tested against each other and their original predecessors that count singletons with evolutionary simulations and/or analyses of a real population dataset for the California seahare (*Aplysia californica*). These tests document that these new approaches offer reliable and fast alternatives for the determination of θ from error prone sequences.

## Results and discussion

### Three new methods for estimating θ from error prone sequences

#### Infinite sites model

As in their original versions, the new Watterson, Tajima, and Knudsen/Miyamoto methods rely on the infinite sites model to accommodate a neutral mutation process [27,28]. The infinite sites model assumes that only a single mutation can occur at any homologous position of the population sample. Thus, each variable site will be represented by only two bases that subdivide the sampled sequences into two non-overlapping subsets consisting of those with the first nucleotide versus the remainder with the second base. Correspondingly, each mutation will map to a specific branch within the sample genealogy, which thereby partitions the sequences into their two non-overlapping subsets. For example, the shared polymorphism at position 3 in Figure 1 is attributable to a unique mutation along the internal branch that partitions sequences I and II from III, IV, and V.

As random errors are treated as rare chance events, they are also modeled in this study along with the mutations by the infinite sites process. Thus, only a single random error or mutation is allowed at any site of the sampled sequences. In turn, each random error is limited to a single sequence (and therefore to a particular singleton) in contrast to a mutation that can also result in a shared polymorphism (Figure 1). The reason is that random errors arise during the experimental determination and recording of individual sequences, whereas mutations occur at specific points within the sample genealogy. Thus, a mutation along an internal branch of the genealogy will result in a new base that will be shared by two or more of its descendant sequences.

#### New Watterson estimator (θ'_*W*_)

Define *T*_*i *_as the length of time (as scaled by *N*_*e *_generations) during which there are exactly *i *ancestors for *n *sampled sequences. Standard coalescent theory tells us that:(1)

and(2)

[4,29,30]. The expected total branch length of the genealogy for *n *sequences (as measured in units of scaled coalescent time) can now be calculated as:(3)

Let *n*_*s *_be the observed number of segregating (polymorphic) sites in the dataset. Under the infinite sites model, *n*_*s *_also counts the number of mutations, since each observed variable site is attributable to a single mutation. The expected number of mutations per locus per unit of branch length is θ/2. Thus, an estimate of θ can be obtained as:(4)

[25].

A new Watterson estimator that avoids singletons (and thereby random sequence errors, θ'_*W*_) can now be derived from Equation (4) by adjusting both its numerator and denominator. The numerator is easily adjusted by counting only the shared polymorphic sites in the original dataset (*n'*_*s*_). Although more complicated, the denominator can also be readily adjusted by including in its calculation only the lengths of the internal branches where shared polymorphisms can arise (see below).

Let us again consider a point in the genealogy where there are exactly *i *ancestors for the *n *sampled sequences. Looking forward in time, the probability that a particular branch of the genealogy is not chosen for the next split (leading to *i *+ 1 lineages) is [(*i *- 1)/*i*]. Thus, the probability that this branch remains unbroken to the present is:(5)

By combining Equations (3) and (5), the total length of the external branches where singletons can occur can now be calculated as:(6)

Our Equation (6) is equivalent to equation (10) of Fu and Li [31], apart from our use of different symbols and terms and of time as scaled by *N*_*e *_generations (rather than generations alone). Thus, as previously noted by them, the total length of the external branches where a singleton can occur is independent of the original number of sampled sequences. Fu and Li [31] also obtained the variance for the total length of the external branches as their equation (14).

**Figure 2:**
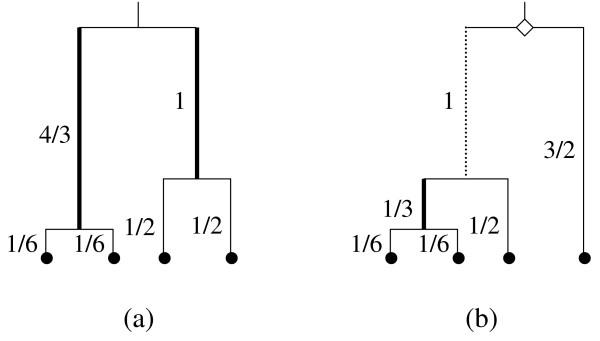
**Representative symmetrical (a) and asymmetrical (b) genealogies for four sampled sequences**. In all, there are six symmetrical and 12 asymmetrical labeled genealogies for these four sequences [8,45]. This figure illustrates how the shape of a genealogy affects whether a mutation will lead to a singleton or shared polymorphism. This heterogeneity contributes to the variance of θ for the three new methods. This contribution is in addition to the heterogeneity of the genealogical branch lengths and Poisson mutation process [4,5]. The diamond highlights the common ancestor of the (*n *- 1) basal group of the asymmetrical genealogy. The dotted and thin solid lines mark the basal branch leading to this ancestor and the external branches, respectively, where a mutation will result in a singleton. The thick solid lines denote the other internal branches where a mutation will lead to a shared polymorphism. Expected branch lengths are given in units of scaled coalescent time next to each internode (those of the symmetrical genealogy are specific for its particular labeled history). Although both genealogies have an expected overall length of 11/3, the total length of the internal branches where a shared polymorphism can arise is 7/3 for (a) but only 1/3 for (b).

In an asymmetrical genealogy with a basal split of one versus (*n *- 1) sampled sequences, a mutation in the internal branch leading to the common ancestor of the (*n *- 1) group will result in a singleton within the dataset (i.e., a variable site where the single nonmember sequence exhibits the unique base) (Figure 2). Thus, the length of this (*n *- 1) basal branch (as weighted by its probability of occurrence) must also be accounted for in the adjustment of the denominator for θ'_*W*_. The weighted length of this (*n *- 1) basal branch can be calculated as follows. First, the chance of this branch is determined as the probability of an (*n *- 1) asymmetrical topology or equivalently as the probability that either one of the two basal branches for the genealogy remains unbroken to the present. According to Equation (5), the latter probability is 2 [1/(*n *- 1)]. Second, the unweighted length of this branch is then calculated as the length of the time interval *T*_2_, which is 1 [Equation (2)]. Thus, the weighted length of the (*n *- 1) basal branch is:(7)

By combining Equations (4), (6), and (7), θ'_*W *_can now be obtained as:(8)

In words, θ'_*W *_ignores the singletons (and thereby random errors) in the original dataset, while restricting the total branch length calculation to only those internal internodes where a shared polymorphism can arise.

Apart from our use of different symbols and terms for calculating the denominator, our Equation (8) is equivalent to equation (13) of Achaz [1]. Achaz [1] also derived his equation B22 for the calculation of the associated variance for *n'*_*s *_(i.e., his Var [*S*_-η1_]).

#### New Tajima estimator (θ'_*T*_)

Let π_*ij *_denote the number of observed pairwise differences between sampled sequences *i *and *j *(for *i *≠ *j*). Standard coalescent theory tells us that the expected waiting time for these two sequences to coalesce is ~ *N*_*e *_generations. Thus, the expected value of each π_*ij *_is θ, whereas that for their sum is:(9)

Rearranging Equation (9) leads to:(10)

[26].

The Tajima and Watterson estimators have the same expected value of θ, even though the former is based on the total differences between each sampled sequence pair whereas the latter is obtained from the total number of segregating sites within the sample. Their different summaries of the observed variation are the basis of Tajima's *D *that tests for departures from the standard neutral model [32] (see also [31,33,34]).

To avoid random errors, Equation (10) can be adjusted in a manner similar to that used to modify Equation (4) of θ_*W*_. First, let π'_*ij *_count the number of observed pairwise differences between sequences *i *and *j *after the removal of all singletons from their dataset. Then, the expected number of singletons can be calculated from Equations (6) and (7) as the total length of the external branches and (*n *- 1) basal branch (as weighted by its probability of occurrence) multiplied by θ/2 {i.e., [1 + (1/*n *- 1)] θ}. The removal of each singleton from the original dataset reduces the expected sum in Equation (9) by (*n *- 1). Combining these results, we obtain:(11)

Rearranging Equation (11) then leads to:(12)

Our Equation (12) for θ'_*T *_is equivalent to equation (15) of Achaz [1], except for our use of different symbols and total π'_*ij *_(rather than average π'_*ij*_) for the population sample. Achaz [1] also derived his equation B23 for the calculation of the associated variance for average π'_*ij *_[i.e., his Var[π_-η1_]).

#### New Knudsen/Miyamoto model (θ'_*KM*_)

Knudsen and Miyamoto ([2], hereafter referred to as "KM") developed a full coalescent/mutation model that accounts for three specific factors of experimental error and design: (a) For random sequence errors, (b) For unobserved polymorphisms due to missing data, and (c) For the uncertain assignment of the multiple sequencing reads for a diploid or polyploid individual to its two or more homologues. Their KM model uses recursion to calculate an exact probability of the population sample under the standard Fisher [35] and Wright [36] model for reproduction (hereafter, referred to as "FW") and the infinite sites process for both mutations and random sequence errors [37,38]. Their model relies on ML to estimate θ and the expected number of errors per full length sequence (ε).

At the heart of the KM model is equation (1) of Knudsen and Miyamoto [2], which is reproduced here as:(13)

The parameters and factors of this equation are defined in Table 1. As one works backwards in time, the probability that the next observed event is a specific coalescence is given by the first term in the top line before the double sum. As indicated by this double sum, if indeed a coalescent event occurs, then it will happen between two sampled and/or ancestral alleles with compatible (if not identical) sequences. Such sequences are referred to as combinable (*s*_*i *_~ *s*_*j*_).

**Table 1:** Definitions of the parameters and factors used in Equation (13) of the KM model

Parameter or factor	Description
*m*_*s*_	The number of segregating sites within the current set of sampled and/or ancestral sequences
σ_*i*_	Counts the number of "singletons" for sampled or ancestral sequence *i*. Here, "singleton" refers both to the derived mutations of the shared polymorphisms for the sampled sequences as well as to those of the observed singletons (in the strict sense) within the original dataset (Figure 3).
*P*_*c*_(α_*i*_(*S*))	Probability of α_*i*_(*S*), which is the current set of sampled and/or ancestral sequences prior to a mutation in sequence *i*
*P*_*c*_(β_*ij*_(*S*))	Probability of β*_ij_*(*S*), which is the current set of sampled and/or ancestral sequences after the coalescence of combinable sequences *i *and *j *(*s*_*i *_~ *s*_*j*_; see below)
*P*_*c*_(*S*)	Probability of *S*, which is the current ordered set of *n *sampled and/or ancestral sequences (*s*_1_, *s*_2_, ..., *s*_*n*_) during a particular coalescent interval in the genealogy
*s*_*i*_, *s*_*j*_	Sampled and/or ancestral sequences *i *and *j *(where *i *≠ *j*)
*s*_*i *_~ *s*_*j*_	Signifies that the available regions of sampled and/or ancestral sequences *i *and *j *are at least compatible and that the two are therefore combinable (i.e., can coalesce)
|*s*_*i*_|	Measures the relative degree to which sampled or ancestral sequence *i *is a complete or partial sequence. Thus, Σ_*i *_|*s*_*i*_| summarizes the total available length of all sampled and/or ancestral sequences during a particular coalescent interval.
|*S*|	Summarizes the current number of sampled and/or ancestral sequences during a particular coalescent interval in the genealogy

**Figure 3:**
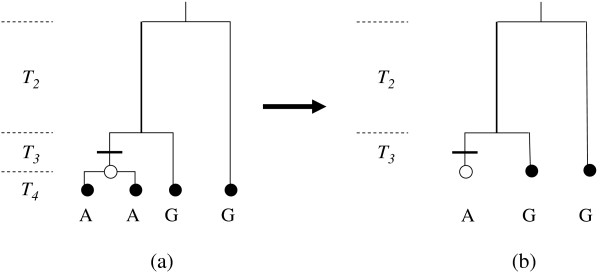
**Illustration of how a shared polymorphism among the sampled sequences becomes counted as a "singleton" as one works backwards in time**. In (a), the A/G bases for a shared polymorphism are shown for four sampled sequences. The three coalescent time intervals of this asymmetrical genealogy (*T*_*j*_, with *j *= 4, 3, or 2 sampled and/or ancestral sequences) are labeled on the left. The solid bar marks the G to A mutation in the common ancestor of the two leftmost sampled sequences (open dot), which results in the A/G shared polymorphism. The first event is the coalescence of the two leftmost sampled sequences in *T*_4 _(a). This coalescence reduces the number of alleles to three as the two leftmost sampled sequences in *T*_4 _are replaced by their common ancestor in *T*_3 _(b). In the process, their shared polymorphism is replaced by the "singleton" of their common ancestor. This replacement is the basis of the expanded definition for a "singleton" (Table 1).

The first term in the bottom line before the single sum is then for the probability that the next observed event is instead a mutation. As indicated by this sum, if indeed a mutation occurs, then it will happen to a sampled or ancestral sequence with at least one "singleton" (σ_*i *_> 0). Here, the definition of a "singleton" is expanded to include the derived mutations of the common ancestors for the different groups of related sampled sequences as well as those for the singletons (in the strict sense) of the original dataset (Table 1). This expanded use of the term acknowledges that a shared polymorphism under the infinite sites model is due to a unique mutation within the common ancestor of those sampled sequences sharing the derived base (Figure 3). Thus, even though they result in shared polymorphisms among the sampled sequences, these derived mutations are ultimately counted as "singletons" as one works backwards in time. This expanded definition of a "singleton" allows for the economical use of σ_*i *_alone to track the mutations of both the original shared polymorphisms and singletons.

**Figure 4:**
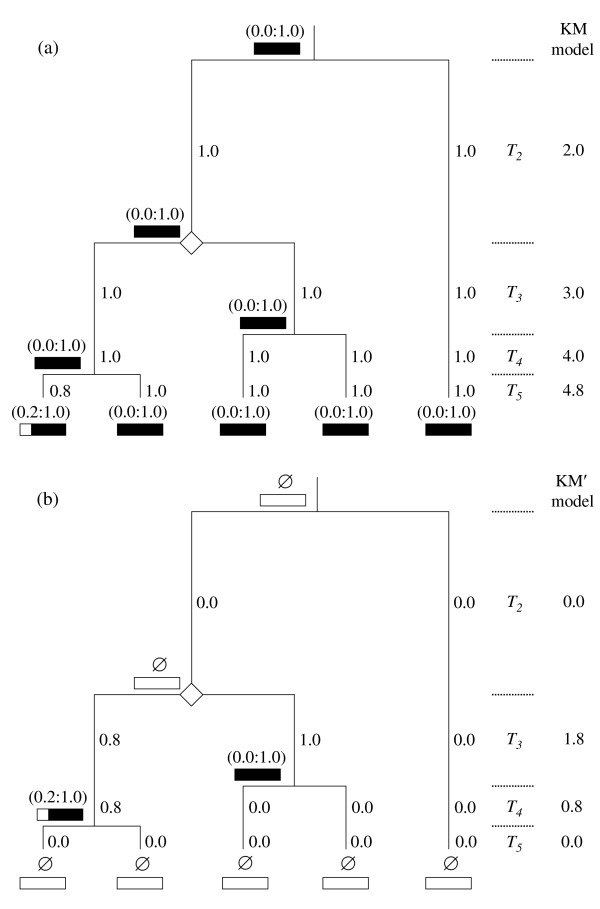
**Genealogy for five sampled sequences illustrating how closed intervals, |*s*_*i*_|, and Σ_*i*_|*s*_*i*_| are calculated by the KM (a) and KM' (b) models**. The closed and open segments of each bar denote the available versus missing or ignored regions of its sequence as scored over the range of (0:1). |*s*_*i*_| are calculated from these scores (numbers next to branches) and Σ_*i *_|*s*_*i*_| is then determined as their sum for each *T*_*j *_(values on the far right). In contrast, the KM' model (b) rescores the sampled sequences and the (*n *- 1) basal ancestral sequence of this asymmetrical genealogy (the diamond) as ∅. Thus, these sequences make no contribution to Σ_*i *_|*s*_*i*_| as their |*s*_*i*_| = 0.0. The KM' model also ignores the first 20% of the sequence for the common ancestor of the two leftmost sampled sequences. This region is missing from the leftmost sampled allele and thereby fails the "below" half of the "above and below" test (see text).

In the KM model, the available region(s) of each sampled sequence is summarized as a closed interval(s) that is scored over the range of (0:1). The |*s*_*i*_| factor then quantifies the total amount of sequence available for this sampled allele. For example, the (0.2:1.0) score for the leftmost (first) sampled sequence in Figure 4a indicates that it is lacking the initial 20% of the full multiple alignment. Thus, |*s*_*i*_| = 0.8 for this partial, leftmost, sampled sequence. In turn, as one works backwards in time, the closed intervals for the available regions of the sequences for common ancestors are calculated as the union of the known lengths for their two immediate descendants. Thus, the closed interval of the common ancestor for the two leftmost sampled sequences in Figure 4a is (0.2:1.0) ∪ (0.0:1.0) = (0.0:1.0), with |*s*_*i*_| = 1.0.

The purpose of Σ_*i *_|*s*_*i*_| in Equation (13) is to track the total available length of all sampled and/or ancestral sequences for the detection of mutations as one works backwards in time. The corresponding use of |*s*_*i*_| in the bottom line of Equation (13) allows for the adjustment of σ_*i *_due to unobserved polymorphisms resulting from missing data.

The KM model uses only Equation (13) when working with error free sequences. In turn, this model also relies on equation (4) of Knudsen and Miyamoto [2] when dealing with error prone sequences. This additional equation of the KM model assumes that the random errors are uniformly distributed along the sampled sequences and that their total number is Poisson distributed with an intensity of λ = ε Σ_*i *_|*s*_*i*_|. The inclusion of Σ_*i *_|*s*_*i*_| in the calculation of λ allows for the adjustment of this error rate to account for incomplete sampled sequences.

In contrast to its predecessor, the KM' model uses only Equation (13) when working with either error prone or error free sequences. As for the new Watterson and Tajima estimators, the KM' model operates by counting only those internal branches of the genealogy where a mutation will result in a shared polymorphism (Figure 4b). The sampled sequences are rescored as unknowns with empty intervals (∅), as is the sequence of the common ancestor for the (*n *- 1) basal group of each asymmetrical genealogy (Figure 2). Correspondingly, |*s*_*i*_| is then reset to 0.0 for these sequences. The KM' model ignores the external and basal branches of the genealogy, where a mutation will result in a singleton, by multiplying σ_*i *_for each sampled and/or (*n *- 1) ancestral sequence by |*s*_*i*_| = 0.0 in Equation (13). In this way, the KM' model discounts the singletons in favor of the shared polymorphisms within the dataset without the use of equation (4) and its associated parameters (e.g., λ and ε) of the original KM model.

For sampled sequences without missing data, the previous explanation is complete as to how the KM' model corrects for the branches of the genealogy where a mutation will result in a singleton. However, for incomplete sampled sequences, the "above and below" test of the KM' model also becomes necessary to correct for the regions of each common ancestral sequence where a mutation in its internal branch will lead to a singleton, rather than to a shared polymorphism, because of this missing information (Figure 4b). In this test, "below" refers to those sampled sequences that belong to the monophyletic group of the common ancestor in question. "Above" then corresponds to the remaining, more distantly related, sampled sequences. In Figure 4b, the two leftmost sampled sequences are direct descendants of the first, leftmost, common ancestor, whereas the other three are not. Thus, these two sampled sequences lie "below," whereas the other three occur "above" the internal branch for this leftmost common ancestor.

The above and below test checks whether comparable information for a region is known for at least two, descendant, sampled sequences below and at least two, more distantly related, sampled sequences above an internal branch in the genealogy. If not, then a mutation within this region of the ancestral sequence that corresponds to this internal branch will result in a false singleton within the original dataset. For example, a mutation within the first 20% of the leftmost common ancestor in Figure 4 will result in a false singleton of the second sampled sequence rather than in a true shared polymorphism of the first and second. The problem is that the leftmost sampled sequence is missing comparable information for the detection of this mutation as a shared change in the leftmost common ancestor. The above and below test corrects for such regions of the common ancestral sequences during the rescoring of their closed intervals and |*s*_*i*_|. As the first 20% of the leftmost common ancestor fails the below half of this test, the KM' model disregards this region during the recalculation of its closed interval as (0:2:1.0) and |*s*_*i*_| = 0.8 (Figure 4b).

### Evolutionary simulations and A. californica dataset

#### Evolutionary simulations

To test the three new procedures, evolutionary simulations were conducted according to standard methods [4]. Two hundred datasets apiece were simulated for eight or 16 sequences of length 500 from a single population under the baseline conditions of the standard FW and infinite sites models with θ = 1, 2, 4, or 8. In addition to these baseline conditions, sequence errors were introduced as four or eight randomly placed changes among the eight and 16 sequences, respectively, for an expected ε of 0.5. Estimates of θ were then obtained for the 200 datasets of each tested combination with the FW model and the original and new Watterson estimators, Tajima estimators, and KM and KM' models.

As expected, the FW model and original Watterson and Tajima estimators overestimate θ when the datasets contain random errors (Table 2). In these cases, the random errors are counted as mutations, thereby inflating their estimates of θ. Furthermore, these overestimations are greater for the Watterson estimator than for the Tajima estimator. The reason is that each singleton makes the same contribution as a shared polymorphism to the number of segregating sites (*n*_*s*_) in Equation (4) of θ_*W*_, but a smaller one to the sum of the pairwise differences in Equation (10) of θ_*T*_. Specifically, each singleton is limited in Equation (10) to the (*n *- 1) pairwise comparisons of the unique sequence to the (*n *- 1) other sequences. Thus, the Tajima estimator is less vulnerable than the Watterson estimator to random errors even though its vulnerability is still significant.

**Table 2:** Results of the evolutionary simulations

Evolutionary simulations			Coalescent/mutation models			Watterson and Tajima estimators			
θ	*n*	ε	θ_*FW*_	θ_*KM*_	θ'_*KM*_	θ_*W*_	θ'_*W*_	θ_*T*_	θ'_*T*_

A1: 1	8	0.0	0.96 ± 0.10	**0.76 ± 0.10**	1.10 ± 0.15	0.98 ± 0.10	1.12 ± 0.16	1.02 ± 0.12	1.10 ± 0.15
A2: 1	8	0.5	*3.49 *± *0.23*	0.90 ± 0.14	0.96 ± 0.15	*2.63 *± *0.1*5	0.94 ± 0.15	*2.04 *± *0.13*	0.95 ± 0.16
A3: 1	16	0.0	1.02 ± 0.10	**0.84 ± 0.09**	1.01 ± 0.12	1.00 ± 0.09	0.99 ± 0.11	0.97 ± 0.10	0.96 ± 0.12
A4: 1	16	0.5	*5.10 *± *0.28*	0.99 ± 0.12	1.02 ± 0.13	*3.43 *± *0.16*	1.03 ± 0.13	*2.01 *± *0.13*	1.01 ± 0.13
B1: 2	8	0.0	1.92 ± 0.17	**1.51 ± 0.17**	1.80 ± 0.25	1.92 ± 0.18	1.81 ± 0.25	1.89 ± 0.20	1.82 ± 0.26
B2: 2	8	0.5	*4.38 *± *0.26*	1.80 ± 0.20	1.92 ± 0.26	*3.44 *± *0.19*	1.91 ± 0.26	*2.91 *± *0.20*	1.92 ± 0.25
B3: 2	16	0.0	2.06 ± 0.16	**1.78 ± 0.15**	2.02 ± 0.19	2.04 ± 0.16	2.04 ± 0.20	2.09 ± 0.19	2.10 ± 0.22
B4: 2	16	0.5	*6.19 *± *0.33*	1.88 ± 0.17	1.92 ± 0.19	*4.32 *± *0.20*	1.92 ± 0.19	*2.95 *± *0.20*	1.96 ± 0.22
C1: 4	8	0.0	-	-	4.05 ± 0.44	4.27 ± 0.35	4.27 ± 0.53	4.31 ± 0.39	4.32 ± 0.54
C2: 4	8	0.5	-	-	3.89 ± 0.46	*5.62 *± *0.33*	4.09 ± 0.53	*5.12 *± *0.38*	4.13 ± 0.54
C3: 4	16	0.0	-	-	4.00 ± 0.31	4.12 ± 0.28	4.21 ± 0.38	4.24 ± 0.36	4.28 ± 0.41
C4: 4	16	0.5	-	-	3.82 ± 0.31	*6.23 *± *0.27*	3.87 ± 0.35	*4.84 *± *0.32*	3.86 ± 0.38
D1: 8	8	0.0	-	-	7.88 ± 0.71	7.75 ± 0.49	7.87 ± 0.78	7.78 ± 0.55	7.85 ± 0.77
D2: 8	8	0.5	-	-	7.90 ± 0.85	*9.67 *± *0.63*	8.38 ± 1.03	*9.22 *± *0.73*	8.39 ± 1.03
D3: 8	16	0.0	-	-	7.82 ± 0.54	8.08 ± 0.49	8.20 ± 0.72	8.26 ± 0.69	8.32 ± 0.82
D4: 8	16	0.5	-	-	8.00 ± 0.58	*10.73 *± *0.52*	8.31 ± 0.71	*9.57 *± *0.68*	8.60 ± 0.81

These results are for the standard FW model (θ_*FW*_) and the original and new Watterson estimators (θ_*W *_and θ'_*W*_), Tajima estimators (θ_*T *_and θ'_*T*_), and KM and KM' models (θ_*KM *_and θ'_*KM*_). They are summarized as the means ± twice their standard deviations for 200 simulated datasets apiece for each of the 16 different combinations of θ, *n *(number of sampled sequences), and ε (A1 to D4). Mean estimates that are significantly greater than or less than the true θ are highlighted in italics and boldface, respectively. No results are provided for the FW and KM models with θ = 4 or 8 (lower left corner), since these calculations are too computationally intensive (see text).

In contrast, the KM model underestimates θ when the sequences are errorless (Table 2). Furthermore, this tendency to underestimate θ is also evident (although not significant) when the sequences contain errors (simulations A2, A4, B2, and B4; see also [2] for additional cases). In these situations, mutations are sometimes counted as random errors, thereby deflating their estimates of θ. In cases where few to no random errors are expected (i.e., finished sequences), one can first perform a likelihood ratio test to determine if ε = 0 [39]. If this null hypothesis cannot be rejected, then the KM analysis should be restricted to only Equation (13). However, if ε > 0 according to this likelihood ratio test, then equation (4) of Knudsen and Miyamoto [2] is also needed and the user of the KM model must remain aware that her/his estimate of θ most likely includes some slight downward bias.

Unlike their original versions, the new Watterson estimator, Tajima estimator, and KM' model all consistently recover the true θ in the simulations both with and without errors (Table 2). Furthermore, these three methods also avoid the tendency of the KM model to underestimate θ due to unnecessary or "greedy" parameters for random errors. In particular, the reliance of the KM' model on one less equation [(4)] and fewer parameters (e.g., λ and ε) makes it much simpler and less prone to over-parameterizations than its predecessor.

The mean estimates of θ for the new Watterson estimator, Tajima estimator, and KM' model are also generally associated with greater standard deviations than those for their original versions (Table 2). The proximal reason for these increased standard deviations is that the elimination of singletons results in the loss of some actual mutations along with the random errors. However, this cost of the three new methods appears to be relatively small, given that their standard deviations are never twice as great as those for their original versions (with these discrepancies usually being much smaller). Indeed, the standard deviations for the three new methods are less than or equal to those of their counterparts in four cases (simulations A2, A4, and B4 for θ'_*W *_and A4 for θ'_*T *_in Table 2). Perhaps more surprising is that the standard deviations for the new Watterson and Tajima estimators are comparable to those for the KM' model, particularly when θ = 1 or 2. These similarities in their standard deviations are surprising given that the KM' model is a full ML model.

As summary statistics, the original and new Watterson and Tajima estimators are all computationally fast, requiring much less than 1 CPU second on a 2.4 GHz Pentium 4 CPU to analyze each of the simulated datasets. More importantly, the KM' model is much faster than the FW and KM models as documented by its completed analyses of the more complex datasets (simulations C1 to C4 and D1 to D4 in Table 2). In contrast, the FW and KM models fail to complete their analyses of these more complex datasets due to time and memory constraints. The KM' model is much faster than the FW and KM models, because it relies on only shared polymorphic sites. Less variable positions results in faster coalescences and fewer choices as one works back through the coalescent/mutation recursion of Equation (13).

#### Aplysia dataset

The *A. californica *dataset was the original motivating force behind the development of the KM model [2]. Thus, this real dataset was also analyzed with the KM' model to test further its performance against that of its predecessor. This dataset consists of 18 sequencing reads for six diploid individuals from a laboratory population of the California seahare at the Laboratory for Marine Bioscience, University of Florida (LL Moroz and AB Kohn, unpublished data). Three cloned inserts were sequenced from each individual as a pair of single sequencing passes starting from both ends of an internal segment of 1731 base pairs for the protein-coding region of the nuclear *FMRF *gene. These pairs of passes overlap in the middle for nine sequences, but at most by only 58 bases. Thus, these 18 essentially single-pass sequences contain many random errors and some missing data and their assignments to the two homologues of each diploid remain uncertain (even though their individual sources are known). Correspondingly, the KM analysis of this dataset with 44 singletons and 10 shared polymorphisms required the full use of its factors for experimental error and design (i.e., for random errors, missing data, and uncertain homologue assignments).

A full ML analysis of this complex dataset by the KM model proved too time and memory consuming and a two step procedure was therefore adopted instead whereby the number of errors was first ML estimated followed by the determination of θ for this ML value [2]. This heuristic approach resulted in an estimate of θ_*KM *_= 6.32 for this sample, which still took more than 12 hours to complete on the same CPU as for the simulations (see above). In contrast, a full ML analysis of this dataset by the KM' model using the same factors for experimental design is much faster as it took less than 1 hour on this CPU to obtain a similar estimate of θ'_*KM *_= 7.17. Correspondingly, nucleotide diversity (π) for this sample is calculated as (7.17/1731 positions) = 0.0041 mutations per site. To summarize, the KM and KM' models both support similar estimates of θ for this real dataset, but the latter (as in the evolutionary simulations in Table 2) is much faster as confirmed by its completed, full, ML analysis.

## Conclusion

Expressed sequence tags, low coverage draft sequences, and other such unfinished products are known to contain random errors due to chance accidents during their data collection and recording [1,11,21,23]. However, such sequences often constitute the only available allelic information for a population and methods to deal with their random mistakes are therefore needed to obtain accurate estimates of θ from these error prone data. This study is based on the simple premise that random sequence errors are distributed as singletons. Thus, one can avoid the random mistakes of error prone sequences by ignoring their singletons in favor of their shared polymorphisms (Figures 2 and 4).

This strategy is implemented in the new Watterson estimator, Tajima estimator, and KM' model. These new methods are all accurate and fast according to their evolutionary simulations and analysis of the real complex dataset for *A. californica *(Table 2). These methods come with the cost of increased standard deviations, but this price appears small or even negligible compared to their advantages of significantly improved accuracy and/or computational speed. Obviously, additional evolutionary simulations and applications to real datasets are now needed to evaluate more fully under what conditions the removal of singletons is warranted in light of this tradeoff. Nevertheless, the current successes with the new Watterson estimator, Tajima estimator, and KM' model support our recommendation that these three methods be given serious consideration when estimating θ from error prone sequences.

Unlike the Watterson and Tajima estimators that represent summary statistics, the KM and KM' models both constitute full ML models that offer the framework for the further incorporation of other experimental and population genetic factors [2]. In particular, such further developments are encouraged for the KM' model in light of its current successes in the evolutionary simulations and *A. californica *analysis (Table 2). For example, biased errors within a sample due to the systematic misreading of specific bases during DNA sequencing, the postmortem biochemical degradation of ancient DNA, and/or other such sources of error can be accommodated by a finite sites process that allows for repeated mistakes as well as mutations at the same sequence positions [14-16,40]. Likewise, additional factors to account for other population genetic processes such as recombination (which is most likely the most important parameter overlooked in this study of the nuclear *FMRF *gene for *A. californica*) can be accommodated by the use of ancestral recombination graphs [41]. Inevitably, these more complex versions of the KM' model will require the use of sampling based procedures for their implementation (e.g., Markov chain Monte Carlo approximations), since the current use of direct ML evaluation will remain practical for only the smaller datasets and simpler models [14,42-44].

## Authors' contributions

BK derived the equations, wrote the computer program for the estimation of θ, and conducted the evolutionary simulations. MMM provided biological interpretations about the results. Both authors contributed to the design and main ideas of this study, wrote and edited the manuscript, and read and approved its final draft.
